# A taxonomy of nursing care organization models in hospitals

**DOI:** 10.1186/1472-6963-12-286

**Published:** 2012-08-28

**Authors:** Carl-Ardy Dubois, Danielle D’Amour, Eric Tchouaket, Michèle Rivard, Sean Clarke, Régis Blais

**Affiliations:** 1 Faculty of Nursing Sciences, University of Montreal, Montreal, Canada; 2 Faculty of Medicine and Health Sciences, University of Sherbrooke, Longueuil, Canada; 3 Department of Social and Preventive Medicine, Faculty of Medicine, University of Montreal, Montreal, Canada; 4 RBC Chair in Cardiovascular Nursing Research, University of Toronto and University Health Network, Toronto, Canada; 5 Department of Health Administration, Faculty of Medicine, University of Montreal, Montreal, Canada

## Abstract

**Background:**

Over the last decades, converging forces in hospital care, including cost-containment policies, rising healthcare demands and nursing shortages, have driven the search for new operational models of nursing care delivery that maximize the use of available nursing resources while ensuring safe, high-quality care. Little is known, however, about the distinctive features of these emergent nursing care models. This article contributes to filling this gap by presenting a theoretically and empirically grounded taxonomy of nursing care organization models in the context of acute care units in Quebec and comparing their distinctive features.

**Methods:**

This study was based on a survey of 22 medical units in 11 acute care facilities in Quebec. Data collection methods included questionnaire, interviews, focus groups and administrative data census. The analytical procedures consisted of first generating unit profiles based on qualitative and quantitative data collected at the unit level, then applying hierarchical cluster analysis to the units’ profile data.

**Results:**

The study identified four models of nursing care organization: two professional models that draw mainly on registered nurses as professionals to deliver nursing services and reflect stronger support to nurses’ professional practice, and two functional models that draw more significantly on licensed practical nurses (LPNs) and assistive staff (orderlies) to deliver nursing services and are characterized by registered nurses’ perceptions that the practice environment is less supportive of their professional work.

**Conclusions:**

This study showed that medical units in acute care hospitals exhibit diverse staff mixes, patterns of skill use, work environment design, and support for innovation. The four models reflect not only distinct approaches to dealing with the numerous constraints in the nursing care environment, but also different degrees of approximations to an “ideal” nursing professional practice model described by some leaders in the contemporary nursing literature. While the two professional models appear closer to this ideal, the two functional models are farther removed.

## Background

Over the last decades, the organization and management of nursing care have come under increased pressures fuelled by convergent forces including cost-containment policies, rising healthcare demands and shortages in the supply of nurses to deliver care. Recently, a number of reports have raised concerns about dysfunctions in nursing care operations resulting from faulty organizational conditions [1-4]. These reports have pointed out the serious and negative impact of poor environments on quality of patient care and ultimately on patient safety. Because nurses are involved in all aspects of service delivery across all healthcare settings, the organization of nursing resources is critical to organizational performance, and managers are being challenged to find operational models of care delivery that maximize the use of available nursing resources while ensuring safe, high-quality care. Nursing care restructuring has resulted in a number of nursing care organization models that vary in terms of staffing patterns, scopes of practice and work environment, among other factors.

Despite the interest in developing new organization models in nursing, little broad-based empirical analysis has been done of the distinctive structures and management processes of models currently in place. Theoretical advances supported by empirical evidence have been limited and have failed to keep pace with dramatic changes in hospital nursing care. The lack of an integrated framework and a systematic approach to assess nursing care organization models has resulted in inconsistent classifications of how nursing care is organized at the unit level [5-8]. Thus, although there is a large body of literature on nursing delivery models, there is very limited empirical or theoretical guidance for organizational redesign. This article is intended to address this gap by presenting a theoretically and empirically grounded taxonomy of nursing care organization models in the context of acute care units in Quebec and comparing their distinctive features. Specifically, we developed a theoretically based framework to capture the diverse components of nursing care organization models and tested it empirically, using data collected at the unit level. The resulting taxonomy provides a new lens that goes beyond the traditional models of nursing care delivery and ensures a more complete and accurate picture of diverse facets of nursing care organization.

## A conceptual framework for developing a taxonomy of nursing care organization models and assessing their impact

Conceptual models have long been recognized as a key instrument in the development of service delivery systems. At a minimum, a conceptual model is a useful organizer to identify a system’s major components and processes, illustrate connections among them and analyze how a structured pattern of resources and processes contributes to the achievement of specific outcomes in a given context [9].

A number of approaches have been described as having potential to ensure nursing care structure is matched to patient care needs for providing safe, high-quality care in a cost-effective manner. Clearly, approaches have been influenced by changes over the years in the discipline of nursing and the culture of healthcare. The classic nursing care delivery models used over the past five decades to describe how nurses deliver care can be defined mainly as allocation systems or personnel assignment systems. Descriptors such as functional, primary and team nursing refer in a general way to the assignment of patient care tasks and responsibilities. In functional nursing, tasks are assigned to nursing and ancillary personnel in accordance with their respective qualifications, based on similar principles to those used in production lines. In team nursing, a small group of healthcare workers with diverse educations, skills/abilities and licensures share responsibility for the care of several patients, working collaboratively under the supervision of the RN team leader. In primary nursing, a single registered nurse is responsible for all care to a limited number of patients for the duration of their hospital stay [10]. These care delivery models mainly reflect the role of the registered nurse (RN) in regard to the patient and other nursing care providers (licensed practical nurses and unlicensed providers). However, such a perspective does not comprehensively depict the organization and context of nursing work. It has been particularly criticized for its narrow focus that does not discriminate clearly among different modes of organizing nursing care. It has been shown that the same designation (primary, functional, or team nursing) has often been applied to units that reflect in reality a variety of different practice models [11,12]. There is also growing evidence that nurses do not always function according to these models and that units are often organized on a more complex basis, reflecting a broader range of organizational attributes [13].

Over the last decade, many new models have emerged in the literature in attempts to better identify the distinctive characteristics of different nursing care organization models. However, many of them are merely variations of the traditional three models (functional, primary and team nursing) or concentrate on specific and limited aspects of nursing practice, neglecting others. Kramer and Schmalenberg [7] have proposed six organizational models (new team, total patient care, modified primary, old team, true primary, varying from day to day) that mostly reflect patient assignment patterns and task allocation strategies, similar to traditional models. Other models such as case management, integrated nursing care and interdisciplinary patient care, focus on organizational attributes that are mainly intended to increase nursing care continuity and interdisciplinary collaboration. Another line of inquiry, pursued by the magnet hospital movement, addresses the organizational attributes that characterize practice environments that some believe promote professional nursing values [14]. Yet another stream of work, driven by concerns about nursing shortages, have focused rather on investigating the associations between staffing levels (coverage and staff mix) and patient outcomes. In staffing research, examination of nursing care organization has usually been confined to nursing workforce characteristics, ignoring management practices and organization of care [15,16].

Thus, despite extensive writing and research activity related to various components of nursing care delivery models, the lack of a unifying theory or of an integrated conceptual framework incorporating the diverse facets of nursing care organization has contributed to a fragmented understanding of nursing care organization. In the increasingly complex context of nursing care and its environment, putting together a comprehensive and realistic picture of nursing practice requires focusing attention not only on who plays what role in the patient care dynamic, but also on factors related to the characteristics of providers and patients, to the setting or context in which care is provided, as well as to the nature of the care provided. These factors are integral aspects of nursing practice that must be considered if we are to fully understand the organizational structure of nursing work, account for processes that produce patient outcomes and identify the factors that influence such processes and consequently the effectiveness of nursing care. Even though the provision of care involves in all cases several groups of providers from diverse disciplines, there is a broad recognition that some outcomes specifically reflect differences in those structural features and processes that define the organization of nursing care. A better understanding of the configuration of factors that define nursing services organization and nursing practice is a potentially useful foundation for better understanding nursing’s contribution to health services outcomes.

For the purposes of this paper, a nursing care organization model is primarily a configuration of key organizational attributes that define a pattern of resources and processes used at the unit level to deliver nursing care. While the purpose of any care organization system is to optimize outcomes for both patients and staff, the ultimate challenge is to open the black box and expose the operant mechanisms through which a nursing care delivery model influences those outcomes. We theorize that outcomes related to nursing care organization models reflect the influence of three organizational correlates that interact with each other: staffing, scope of practice and work environment. The combination of these three characteristics is an attempt to integrate three distinct streams of research—nursing care administration, economics of nursing care and human resource management—to build a foundation for examining the complex features of existing and newly emerging nursing care organization models. Taken together, these dimensions address the types of workers providing nursing care and how they are deployed, the content of their work and the context in which they provide services. We illustrate below how these conceptual characteristics provide a theoretically grounded framework for meaningful representation of underlying structures and processes associated with nursing care organization at the point of patient care delivery.

**Figure 1:**
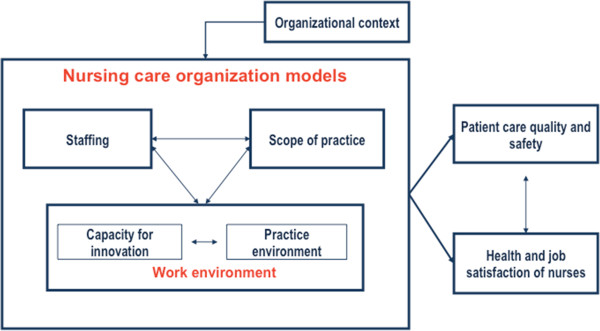
A nursing care organization framework.

Our model (Figure 1) posits that a nursing care delivery model is a specific configuration of three conceptual dimensions (nurse staffing, nurses’ scope of practice and nursing work environment) that interact dynamically to influence both nurse outcomes (health and job safety of nurses) and patient outcomes (patient care quality and safety). Nursing work environment is measured by considering two aspects: the practice environment and the capacity for innovation.

### Staffing

Considerable evidence suggests that nurse staffing has important associations with outcomes of care and that staffing levels are a necessary but not sufficient condition for favorable patient outcomes. Theoretical works have highlighted staffing as a key parameter in the organization of nursing care. The purpose of any delivery system is to provide high-quality care efficiently and effectively. In a labor-intensive sector such as nursing care, maintaining effective staffing levels and skill mixes is a core requirement. Theoretical approaches that emerge from Donabedian’s structure, process and outcomes models or that expand on that framework by integrating additional dimensions (outcomes models, Nursing Role Effectiveness Model) recognize, in all cases, the importance of staffing as a major structural characteristic of nursing care [17-20]. Staffing, an essential feature of nursing care organization, is the process of determining the appropriate number, types and mix of nursing resources to meet workload demands for nursing care at the unit level. It reflects the extent to which the nursing system obtains an adequate supply of staff to achieve its objectives.

### Scope of practice

Beyond structural factors focused on the characteristics of nursing resources and their mix, another major factor in our framework is the type and scope of services provided by nurses. This aspect of nursing care organization is often described in the literature in terms of nursing care processes or nursing work content and refers to activities in which nursing staff engage to deliver care to patients and their families [21]. It indicates what nurses do for, with or on behalf of patients in their daily work, which encompasses the scope of nursing interventions for each domain of care and category of processes. From this perspective, the nurse’s scope of practice is a significant component of any nursing care organization model and includes not only the provision of comprehensive care that meets patients’ needs, but also the extent to which job design enables nursing staff to use the full extent of their professional knowledge and skills and to cover their whole practice domain. The integration of scope of practice into our framework is an attempt to fill a gap in the existing literature. Although the above-mentioned theoretical frameworks [17-20] attest to the importance of work content as a core feature of nursing care organization, this importance has been consistently ignored in operational definitions of nursing care organization models, due in part to having few reliable and valid measures to adequately evaluate the diverse dimensions of nursing work content.

### The work environment

The environment in which nurses provide services is another key dimension we and others use to characterize nursing care organization. The human resources management literature suggests that workers and their work environment are reciprocally related, each influencing the other in an ongoing, dynamic interplay that affects the nature of the work and the outcomes [22-25]. This means that appropriate delivery of nursing care not only calls for investments in staffing and decisions related to scope of practice, but also the purposeful creation of practice environments that enable available staff to perform their work [26]. Conceptually, such interventions encompass the physical, cognitive, psychosocial and professional dimensions of the work environment that support nursing practice [27,28]. Some feel that magnet hospitals are embodiments of these dimensions, which include nurse participation in institutional decision-making at the highest levels of management, flat organization of the nursing service with decision-making decentralized to the nursing unit, substantial autonomy for nurses and independence in their role, collegiality between nurses and other professional groups, and support of nurses from co-workers and administration [5,29,30]. All these characteristics are considered to be conditions that facilitate nursing professional practice and are measured by the Nursing Work Index (NWI), an instrument that has been extensively used to assess nurse practice environments. However, there is widespread consensus that the dimensions tapped by the NWI do not exhaustively capture all relevant aspects of nursing context. Based on a systematic evaluation of existing instruments on nursing practice environment and a synthesis of the literature, Lake [30] concludes that current instruments should be supplemented to ensure a more comprehensive coverage of salient domains related to nursing work context. Our proposed framework expands the coverage to include capacity for innovation in the work setting. In the dynamic environment of nursing care, it is essential that the nursing production system have innovative capacity to ensure nursing staff can adjust constantly to address patient needs and deliver safe, effective, patient-centered, timely and efficient care. A vast empirical study by the Robert Wood Johnson Foundation involving in-depth examinations of 24 care delivery models has revealed that innovative practice environments are an essential feature of many emerging nursing care delivery models. Innovative practice environments are defined as those in which nurses at the unit level are empowered and have access to the levers needed to carry out new roles, smooth patient transitions, foster patient involvement in nursing care and monitor the impact of their work on quality, safety and cost [31]. In addition to those aspects that generally define magnet hospitals, examining innovative capacity at the unit level makes it possible to take into account a broader set of factors, such as effective leveraging of nurses’ role, technology, linkage with the community, results management and feedback, and patients’ centrality in care.

Thus, drawing on several streams of research and knowledge, our proposed framework reflects a more integrated approach to nursing care organization models that takes into account three key organizational attributes: staffing patterns, scope of practice and work environment. As such, this framework ensures a deeper understanding of systems of nursing care provision at the point of care in healthcare organizations. Rather than isolating the three attributes, this integrated framework recognizes relationships among them and provides a theoretical basis for examining configurations of these organizational characteristics. Using this framework, this research had two main objectives: 1) to identify and classify the types of nursing care organization models most commonly used in acute care units in Quebec; and 2) to describe and compare the distinctive features of those models based on a range of data that account for key aspects of nursing care.

## Methods

The methods for this study followed the classical steps of empirical development of taxonomy and were designed to identify clusters of units sharing common features.

### Sampling approach

Twenty-two acute care medicine units located in 11 hospitals in Quebec were selected. While this sample cannot be considered exhaustive of all possible or probable forms of nursing services organization, nonetheless, the sample selection was informed by a preliminary survey of 50 hospitals. Information collected for each hospital included its teaching status, nurse staffing patterns and the degree of implementation of a newly-developed clinical nurse role, in the form of positions reserved for university-trained professional nurses that involve responsibility for care coordination, team leadership and coordination of professional development activities in addition to the provision of direct care. Our intent was to sample from settings having adopted a broad range of organization models in designing nursing care. The final sample reflected a diverse mix of units selected from organizations that varied on a range of characteristics: teaching status (university and community hospitals), size, location (urban, suburban, rural), nursing workforce profiles (diverse rates of nurses with university degrees) and varying levels of activity in terms of work reorganization.

### Data collection

To assess the characteristics of nursing care organization in the selected units, our data collection included the following procedures.

A nurse survey. A questionnaire was sent to all registered nurses working as regular employees in the 22 selected units. It was completed by 285 nurses (a 55% response rate), which compares favorably with rates seen in other voluntary surveys of health professionals. The survey was designed to provide information on two specific facets of nursing care organization: scope of practice and nurses’ perception of their practice environment. To assess the profile of the nursing workforce sample, the questionnaire included questions related to demographic and socio-professional attributes, including age, sex, employment status (part-time/full-time), current position title, educational qualifications, years in current position, years of experience as a nurse, and work shift.

Interviews and focus groups with staff nurses and managers. Focus groups were conducted in each of the 22 selected units with groups of four to seven nurses. These group discussions were used to collect information on one aspect of the work environment, which was the capacity for innovation at the unit level. They also provided information about overall unit characteristics, including team composition, work division, work climate, type of care, clients’ characteristics and availability of technologies (information technology, drug delivery systems, basic equipment, and other clinical tools such as therapeutic nursing plans). We also conducted 66 semi-structured interviews with key informants including head nurses, human resources officers and CEOs. These interviews provided insights into various aspects of nursing care at the organizational level: staffing policies, resource availability, human resources practices, nursing influence on decision-making, and capacity for innovation.

A daily census. This census, conducted over 30 consecutive days, collected detailed administrative data on staffing and bed occupancy on each of the 22 selected units.

### Measurement of variables

#### Staffing

Staffing was measured using two indicators, staffing intensity and staff mix, that reflect the concentration of nursing staff caring for patients and the composition of the nursing team, respectively. Staffing intensity measured the quantity of nursing resources available for nursing care and was calculated as a ratio of nursing hours per patient day for all staff types, regardless of the licensure level or the shift (day, evening, night) worked. Nursing hours referred to the total number of direct payroll productive hours from RNs, licensed practical nurses (LPNs) and orderlies. The measure also included hours of care provided by agency or float team nurses and overtime hours. However, nurse manager and clinical specialist hours were not included in these statistics intended to capture staffing levels in relation to direct care delivery. Nursing staff mix was calculated as the proportion of hours provided by registered nurses in the total staffing hours (RNs, LPNs and orderlies combined).

#### Actual scope of nursing practice

The scope of practice, as measured in this study, referred to the extent to which professional nurses’ day-to-day work activities reflect the full range of involvement in care and professional practice development for which they have been educated. It encompasses the full range of functions, responsibilities and activities that a nurse performs in delivering patient care and leadership in relevant ways. Thus the focus was on nurses’ actual or “real” practice in their current jobs and how they carry out their roles in the context of their daily practice, where their work is subject to a variety of influences including employer policies, available resources and technologies [32-34].

For this study, we developed a questionnaire, the ASCOP (The Actual SCOPe of Nursing Practice), using a systematic process that included literature review, expert validation and pretesting. The final version of the instrument included 26 questions that covered six key domains of nursing care: planning and evaluation; patient/family education; communication and coordination; staff orientation and mentoring; quality and safety; and knowledge utilization. Nurses were asked the extent to which they carried out specific activities related to each domain in their daily work. Responses were ranked on a 6-point Likert-type scale (1 = never, 2 = very rarely, 3 = sometimes, 4 = frequently, 5 = almost always; 6 = always). The activities selected in each domain were not intended to describe the whole range of nursing activities, but rather to cover the breadth and depth of the nursing work continuum. The development and the psychometric validation of the ASCOP are described in detail in a separate article [35]. Results support the use of the ASCOP as a reliable and valid tool to measure nurses’ actual scope of practice. The instrument has good internal consistency, with an alpha coefficient of 0.89 for the instrument as a whole and between 0.61 and 0.70 for individual dimensions. Construct validity, assessed using principal component analysis (PCA), showed high percentages of explained variance. Indeed, the six dimensions taken together explained nearly 59% of the scope of nursing practice; the individual dimensions explained between 40% and 62% of the variance.

#### Work environment

In this study, two distinct concepts were measured under the broader category of work environment: the nursing practice environment and the capacity for innovation.

The nursing practice environment is defined by work settings’ organizational characteristics that either facilitate or constrain professional nursing practice. Such attributes are believed to be associated with the establishment and maintenance of supports that enable regular and sustained high-quality professional nursing practice. To measure this aspect of the work environment, we used the Practice Environment Scale of the Nursing Work Index [29]. Subscales included: nurse participation in hospital affairs; nursing foundations for quality of care; nurse manager ability, leadership and support of nurses; staffing and resource adequacy; and collegiality of nurse-physician relations.

The capacity for innovation refers to the extent to which the practice environment empowers nurses to play their role as change agents and enables them to improve their practice. This was assessed with qualitative data collected from nurses and managers at the unit level, using five indicators derived from a large American study [31]. These indicators define five main features of innovative nursing care delivery models: elevated RN role; sharpened focus of care on the patient; mechanisms to ensure smooth patient transitions across care settings and links with communities; mechanisms for monitoring results to improve performance; and leveraging of technologies to optimize work processes. Classification of each unit on these five indicators was conducted systematically. First, for each indicator, several tracers were identified from the literature survey (see example in Table 1). The resulting template was used to rate the different units. The rating scale offered three options: 0 to indicate that the tracer was absent in the unit; 1 to indicate evidence of tracer presence; and IO (insufficient information) to indicate that tracer presence or absence could not be assessed from available information. An aggregate score was calculated for each indicator based on available information, and each unit was then ranked (high or low score) for each of the five indicators. To improve construct validity, the template was initially tested on two pairs of units by two pairs of reviewers. Based on their feedback, the tool was modified slightly to refine the description of some items. To assess the reliability of the exercise, each unit was independently rated by two reviewers. The interrater reliability score reached 96% for the overall exercise. 

**Table 1:** Example of the operationalization of one of the five indicators used to measure the capacity for innovation

**Availability of technologies (4 items)**	- Availability of advanced information technologies (computerized nursing chart);
	- Automative drug distribution system (e.g. Pyxis));
	- Therapeutic nursing plan implemented and widely used;
	- Availability of basic equipment (patient lift; electric beds, alternating-pressure mattresses, sphygmomanometers)
	*This marker was rated “strong” for a unit when three of the four identified tracers were assessed as being present in the unit based on interview transcripts*

### Data analysis

The analytic approach to taxonomy development is well established in the organizational literature [36] and has been applied to healthcare in a number of prior studies [37-39]. In this study, the unit of analysis was the care unit. The 22 units were grouped into nursing care organizational models using SPAD (Software for Predictive Analysis and Data Mining). Based on qualitative and quantitative data collected from nurses and managers at the unit level, all units were first rated for all those characteristics or factors that were selected to describe and group them. Hierarchical cluster analysis was then applied to the units’ profile data that consisted in grouping individual cases into increasingly larger clusters while ensuring both homogeneity of cases within each cluster and heterogeneity across the identified clusters. The analytical steps were as follows:

Data reduction. A first step in this analysis was to define the organizational characteristics or factors that would be used to group the 22 units. Because the instruments used to operationalize the four components of the framework included a large number of variables, we aimed to reduce the dimensionality of each component while retaining as much variation as possible. This procedure was particularly needed for the scope of practice measurement tool and the Nursing Work Index (used to measure the practice environment). The objective at this step was to describe the units’ profiles for each component of the framework and rank them based on their profiles.

#### Units’ profiles with regard to actual scope of nursing practice

Data were first aggregated at the unit level, and the 22 selected units were rated for all indicators of each dimension. Using PCA (principal component analysis), we reduced the six indicators used to assess scope of practice to two factors: 1) evaluation, planning and patient education; and 2) training and quality. These factors combined indicators that explained most of the variation among the different units. For each factor, an aggregate score was calculated for each of the 22 units. This score was then converted from continuous data to categorical data and the units were ranked into three categories: more broad, moderately broad and less broad scope of practice. The class intervals were defined on the basis of the statistical distribution of scores observed among the 22 units to ensure homogeneity within each class. Table 2 presents a matrix of the units’ profiles for the two actual scope of practice factors and the resulting classification of the units for this dimension.

**Table 2:** Summary of profiles for “actual scope of practice” and “practice environment”

		**Actual scope of practice**			**Practice environment**		
**Hospital Code**	**Unit Code**	**Evaluation, planning and patient education** ***(factorial scores level)***	**Training and quality*(factorial scores level*)**	Unit ranking for actual scope of practice*^,^**	**Support to professional practice (*factorial scores level)***	**Physician-nurse collaboration*(factorial scores level)***	Unit ranking for practice environment*^,^**
A	1	low	high	moderately broad	low	low	less supportive
	2	medium	medium	moderately broad	low	medium	less supportive
B	3	high	low	moderately broad	high	high	more supportive
	4	high	high	more broad	high	high	more supportive
C	5	low	high	moderately broad	high	high	more supportive
	6	low	medium	less broad	medium	high	more supportive
D	7	high	high	more broad	medium	low	less supportive
	8	medium	high	more broad	low	medium	less supportive
E	9	medium	high	more broad	medium	low	less supportive
	10	medium	low	less broad	medium	medium	moderately supportive
F	11	low	high	moderately broad	low	low	less supportive
	12	medium	high	more broad	medium	medium	moderately supportive
G	13	low	medium	less broad	medium	high	more supportive
	14	medium	medium	moderately broad	low	medium	less supportive
	15	high	low	moderately broad	medium	medium	moderately supportive
H	17	low	low	less broad	medium	low	less supportive
	24	low	low	less broad	medium	medium	moderately supportive
I	18	high	low	moderately broad	high	medium	more supportive
	19	medium	medium	moderately broad	high	high	more supportive
J	20	high	medium	more broad	medium	medium	moderately supportive
	21	low	low	less broad	medium	medium	moderately supportive
K	22	low	low	less broad	low	medium	less supportive

*Less (broad or supportive) **⇒** low score on two factors, or low score on one factor and medium score on the other.

Moderately (broad or supportive) **⇒** medium score on two factors, or low score on one factor and high score on the other.

More (broad or supportive) **⇒** high score on two factors, or medium score on one factor and high score on the other.

**Using the unit with the highest score as reference, high scores included the units with a score in the range of 60-100% of the reference unit, medium scores ranged from 45% to 59%; low scores were all those below 45%.

#### Units’ profiles with regard to practice environment

The same procedure described above for scope of practice was used to reduce the six dimensions of the practice environment down to two factors: support to professional practice and physician–nurse collaboration. Based on scores for those two factors, the units were ranked into three categories: more supportive, moderately supportive and less supportive practice environment. Table 2 presents a matrix of the units’ profiles for the two practice environment factors and the resulting classification of the units for this dimension.

#### Units’ profiles with regard to staffing

Two measures were obtained for each unit: average rate of nursing hours per patient day (staffing intensity) and average percentage of RN worked hours in the total nursing hours. No data reduction was applied, and the 22 units were assigned to one of two categories (high or low) for both of the staffing dimensions. Table 3 presents a matrix of the units’ profiles for the two indicators related to staffing and the resulting classification of the units for this dimension.

**Table 3:** Summary of profiles for “staffing” and “capacity for innovation”

		**Staffing**			**Capacity for innovation (scores by dimension and unit ranking)**					
**Hospital code**	**Unit code**	**Percentage of worked hours by RN**	**Number of nursing hours per patient day**	Unit ranking for staffing*	**Elevated RN role**	**Focus of care on the patient**	**Patient transition**	**Focus on results**	**Leveraging of technologies**	Unit ranking for capacity of innovation**
A	1	low	low	few nurses and few hours	low	low	high	low	low	less innovative
	2	low	low	few nurses and few hours	low	low	low	low	low	less innovative
B	3	high	low	many nurses and few hours	low	high	high	low	low	moderately innovative
	4	high	low	many nurses and few hours	low	high	high	high	low	moderately innovative
C	5	high	low	many nurses and few hours	low	low	low	high	low	less innovative
	6	low	low	few nurses and few hours	low	low	low	high	low	less innovative
D	7	low	high	few nurses and many hours	low	low	low	high	high	moderately innovative
	8	low	high	few nurses and many hours	low	low	low	high	low	less innovative
E	9	low	high	few nurses and many hours	low	low	low	high	low	less innovative
	10	high	high	many nurses and many hours	low	high	high	high	low	moderately innovative
F	11	low	high	few nurses and many hours	low	low	high	low	low	less innovative
	12	low	high	few nurses and many hours	low	low	low	low	low	less innovative
G	13	high	low	many nurses and few hours	high	high	high	high	high	more innovative
	14	high	low	many nurses and few hours	low	low	low	low	low	less innovative
	15	high	low	many nurses and few hours	high	low	low	low	low	less innovative
H	17	low	low	few nurses and few hours	low	high	high	high	high	more innovative
	24	low	low	few nurses and few hours	low	low	low	low	low	less innovative
I	18	high	high	many nurses and many hours	high	high	high	high	high	more innovative
	19	high	high	many nurses and many hours	high	high	high	low	high	more innovative
J	20	low	high	few nurses and many hours	high	high	high	low	high	more innovative
	21	low	high	few nurses and many hours	low	high	high	high	low	moderately innovative
K	22	low	high	few nurses and many hours	low	high	low	high	low	moderately innovative

* “Few nurses” **⇒** Proportion of RN hours in total nursing hours < 47%.

“Many nurses” **⇒** Proportion of RN hours in total nursing hours ≥ 47%.

“Few hours” **⇒** Hours of care per patient day < 6.

“Many hours” **⇒** Hours of care per patient day ≥ 6.

**less innovative **⇒** high score on one indicator or less.

moderately innovative **⇒** high score on two or three indicators.

more innovative **⇒**high score on at least four indicators.

#### Units’ profiles with regard to capacity for innovation

Each unit was first rated (low or high score) on each of the five indicators. Then the units were ranked into three categories: more innovative (high rating on at least four indicators), moderately innovative (high rating on two or three dimensions) and less innovative (high rating on one dimension or less). Table 3 presents a matrix of the units’ profiles for the five indicators related to capacity for innovation and the resulting classification of the units for this dimension.

Defining the factorial axes. A second step of the analysis was to define the groupings of characteristics that most differentiated the selected units. This analytical step was achieved through multiple correspondence analysis, using ranked data obtained in earlier phases. Thus, the four variables used for these groupings were the unit rankings for each of the four dimensions of the conceptual framework. The use of these four categorical variables was based on the type of analysis performed and the imperative of minimizing the number of variables, taking into account the sample size (22 units). The groupings of characteristics resulting from this analysis formed a series of factorial axes. Two criteria were used to specify the number of factorial axes to be retained: the degree to which the characteristics included in one factorial axis satisfactorily described each of the conceptual dimensions, and the additional contribution made by adding characteristics from another factorial axis [39]. In our study, the first three axes (explaining 58.02% of the total variation) were used to group the 22 units into models of nursing care organization.

Partitioning the selected units into homogeneous subgroupings. For each factorial axis, SPAD calculated a factorial score for each unit and then grouped the units based on their factorial scores using ascending hierarchical clustering. The number of partitions was chosen so as to maximize homogeneity within a class (intraclass inertia index) and maximize heterogeneity among classes (interclass inertia index). Based on these indexes, the organizational characteristics of the cases observed were best described by grouping them into a four-cluster solution characterized by 27.16% intraclass inertia and 62.84% interclass inertia. Such levels for these indexes indicate that the four-class partition represents an acceptable compromise to maximize homogeneity within each group and maximize the heterogeneity among the four groups [40]. It can be seen in Table 4 that the alternative option of a three-cluster solution would have resulted in less discriminant groupings, while the five-cluster solution would have resulted in more groups and a decrease in their internal homogeneity. 

**Table 4:** Intraclass and interclass inertia for three alternative options of cluster solution

**Number of clusters**	**Intraclass inertia**	**Interclass inertia**
3	54,30%	45,70%
4	27,16%	62,84%
5	30,96%	69,04%

Defining the distinctive organizational characteristics for each model. The last step, as indicated above, was to define the distinctive characteristics for each cluster. To this end, SPAD provided, for each characteristic, a value test that indicated the extent to which this characteristic was shared by all cases in the group. For this study, a characteristic was used in the model only if its value test was higher than 1.7 (with a p-value threshold of 10%). This threshold is appropriate for samples’ size in the range of this study (22 units) because of the risk of rejecting the true hypothesis with a p-value threshold of 5% [40,41].

### Ethics

This study obtained approvals from two research ethics committees: 1) the research ethics committee of the University of Montreal; and 2) a multicentre research committee led by the *Centre hospitalier de l’Université de Montréal* and involving all the local institutional research ethics committees in the 11 hospitals where data were collected.

## Results

This section presents the taxonomy resulting from the data analysis and key distinguishing features associated with the different clusters.

### Four nursing care delivery models

**Figure 2:**
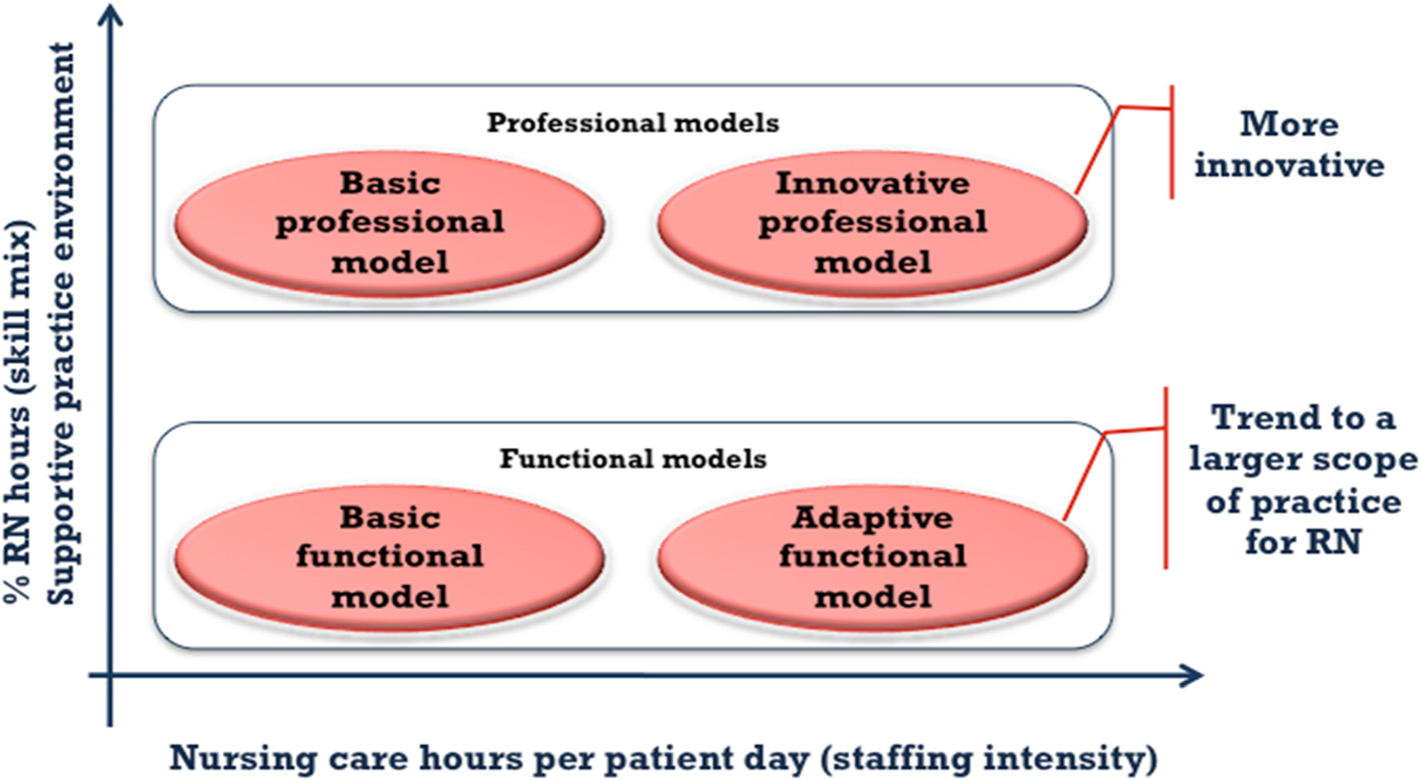
**Four nursing care organization models.**

As described in the previous section, a four-cluster solution emerged as the most stable for grouping the units. This taxonomy distinguishes four approaches to organizing nursing care delivery. Two models are defined as professional, as described in earlier works [42,43]. From this perspective, nursing is recognized as a professional practice primarily reserved for people with a certain level of education, and healthcare organizations are engaged in developing professional structures that support the efforts of these knowledge workers, who exercise considerable discretion in carrying out their work. The other two are defined as functional. This refers to a view of nursing as a broad set of tasks that can be done by a variety of workers, and the focus for healthcare organizations is to subdivide work among many workers, use them flexibly and control their activities (See Figure 2). Table 5 presents the distribution of the 22 units among the four models. 

**Table 5:** Distribution of the 22 units among the four models

**Model**	**Hospital categories**	**Number of units**
Basic professional model	1 university hospital, 1 university affiliated hospital, 1 community hospital	6
Innovative professional model	1 university affiliated hospital	2
Basic functional model	3 community hospitals	5
Adaptive functional model	1 community hospital, 2 university hospitals, 2 university affiliated hospitals	9

### Professional models

The professional models draw mainly on registered nurses as professionals to deliver nursing services and reflect a stronger support to nurses’ professional practice. They are characterized by a higher proportion of care hours provided by registered nurses and by nurses’ perception of enjoying greater support for their professional practice. These models were observed, except for one case, in units located in university or university-affiliated hospitals and appeared to reflect efforts invested, to varying degrees, to ensure that a certain proportion of nursing care be directly delivered by registered nurses despite economic and labor constraints.

We identified two professional models with distinctive features: the basic professional model and the innovative professional model.

The basic professional model was observed in six of the 22 units and was characterized by: a relatively high proportion of hours of care provided by registered nurses (63%); a low intensity of staffing, as reflected in the hours of care per patient day (3.9 to 4.9 hours); nurses’ more positive perception of their practice environment; a moderate level of nurses’ actual scope of practice; and a few features emblematic of innovation.

The innovative professional model was observed only in two of the 22 units. Its key distinguishing features were: a higher proportion of hours of care provided by registered nurses (73%) combined with a high intensity of staffing resources (6.5 to 7.7 hours of care per patient day), as well as a high level of innovation tracers. This was also the only model that featured a relatively high level of nursing care provision by university-trained nurses, that is, RNs with university training, at 28% of hours of care compared to 5 to 9% in the three other models. This model did not differ from the previous one with regard to nurses’ actual scope of practice, which remained at a moderate level. As in the basic professional model, nurses working in this model perceived a more supportive practice environment than those working in the following two models, even though the practice environment scores were not optimal.

### Functional models

The functional models draw more significantly on LPNs and assistant staff (orderlies) to deliver nursing services than do the professional models. They seem to reflect the search for strategies to deal with economic and labor-market constraints and to use nursing resources flexibly. They are characterized by a lower proportion of hours of care provided by registered nurses, and nurses’ perception that the practice environment is less supportive of a “professionalized” approach to the work of registered nurses. Two models fall into this category: the basic functional model and the adaptive functional model.

The basic functional model was observed in five of the 22 units, all in community hospitals. These units were characterized by a low proportion of hours of care provided by RNs (46%), a low intensity of staffing (4.5 to 5.5 hours of care per patient day); nurses’ perception of a relatively low level of support from their practice environment; and low levels of both nurses’ actual scope of practice and innovation tracers.

The adaptive functional model was observed in nine units and was clearly distinguished from all the others on two features: higher staffing intensity (6 to 8.3 hours of care per patient day), driven essentially by increased use of LPNs, and a trend toward a larger scope of practice for registered nurses. Five of the six units that had the highest rates for nurses’ actual scope of practice were included in this model. The observed rates for the practice environment and for innovation tracers were both low, at levels close to the basic functional model.

Table 6 summarizes the main features of the different groups of units included in each model and the differences among them.

**Table 6:** Main features of the different groups of units included in each model with regard to the dimensions of the conceptual framework

			**Innovative professional model**	**Basic professional model**	**Adaptive functional model**	**Basic functional model**
Staffing		Average hours of care per patient -day	6.62	4.26	7	5
		Average proportion of RN hours in total nursing hours (%)	73	63	46	46
		Average proportion of university graduate nurse hours in total nursing hours (%)	28	9	5	7
		Average proportion of LPN hours in total nursing hours (%)	1,5	9	21	24
Work environment	Innovation	Average score for capacity for innovation (maximum possible score of 5)	4.5	2	1.9	1.2
	Practice environment	Average score for practice environment (maximum possible score of 4)	2.75	2.56	2.39	2.33
		Number of units with high score for practice environment factors/total number of units in the model	2/2	4/6	0/9	1/5
		Number of units with moderate score for practice environment factors/total number of units in the model	0/2	1/6	4/9	1/5
		Number of units with low score for practice environment factors/total number of units in the model	0/2	1/6	5/9	3/5
		Average score for scope of practice (maximum possible score of 6)	3.52	3.49	3.37	3.29
		Number of units with high score for scope of practice factors/total number of units in the model	0/2	1/6	5/9	0/5
		Number of units with moderate score for scope of practice factors/total number of units in the model	2/2	4/6	1/9	2/5
		Number of units with low score for scope of practice factors/total number of units in the model	0/2	1/6	3/9	3/5

## Discussion and conclusion

Our work in this study was based on a conceptual framework focusing on three key components—staffing, nurses’ actual scope of practice, and work environment—to describe nursing care organization models in hospitals. The framework was built on different streams of research and theory, in the hope that the synthesis can capture the complex features of constantly changing nursing care settings better than did earlier, narrower approaches. Much that has been written about these models reflects a narrow focus (staffing, task assignment, patient allocation between providers) and has contributed to a fragmented understanding of nursing services delivery. Our framework is intended to enhance existing descriptions of nursing practice by including a more comprehensive set of factors that covers both structural characteristics and organizational processes. Nursing care organization is thus conceptualized as a multidimensional process that goes beyond the traditional focus on staff mix to bring attention to the actual work performed by nurses at the point of care and to their work environment. Another important feature of this framework is its relational structure. As such, it provides a theoretical basis for better understanding the complex set of interrelated elements and relationships that define nursing service organization. For managers, policy-makers and health executives, the proposed framework offers new insights into key components that shape the organization of nursing services at the point of care and provides a valuable tool to guide the design of organizational interventions aimed at improving nursing care organization.

The organizational taxonomy that was ultimately developed depicts a number of different configurations of staffing, work organization, and care environment seen in a specific type of care setting—the medical inpatient unit. Despite similar performance-oriented claims, this taxonomy shows that models of nursing care organization do not invariably follow the same path. The findings in Table 6 confirm striking differences in how nursing care is organized in different hospital units and the complexity of diverse ways of mixing nursing resources, using the skills of nursing staff, designing work environments and supporting innovation. Each is the end result of multiple decisions to deal with the numerous constraints and pressures in the nursing care environment such as shortages of nurses, increased acuity of patients’ conditions, and budgetary constraints. However, none of the four models emerging from this taxonomy reflects the exact characteristics of the ideal of nursing professional practice models as it has evolved in the contemporary nursing literature [14,44-46]. Rather, the four models show different degrees of attainment of this ideal. While the innovative professional model and, to a lesser extent, the basic professional model appear closer to this ideal, the two functional models are farther removed. Common to both professional models are staffing systems that rely mostly on registered nurses to deliver nursing care and on work environments that are more supportive to professional nursing practice. The enabling conditions associated with the work environment are particularly marked for the innovative professional model. However, the research results show that, even for the professional models, the scores on several dimensions are far from optimal. As an example, the current scope of nursing practice evidenced in these models suggests that nurses’ practice does not incorporate the full range of activities for which professional nurses are prepared by their educations or in which they are legally allowed to engage. Similarly, nurses’ perceptions of their work environment suggest there is still room for improvement on several fronts.

The unit groupings reflect the contextualized nature of nursing care organization models and their managerial influences. Except in one case, the two units in each hospital were classified identically in the grid. The five units forming the basic functional model were all in community hospitals, suggesting that these types of organizations may be faced with more severe resource constraints that are ultimately reflected in their staffing and care delivery models. Furthermore, the two units forming the innovative professional model were also in the same university-affiliated hospital that has a long tradition of professional focus in nursing care delivery and has long been characterized by efforts to recruit university-trained nurses, promote a professional culture and support professional development. Overall, the findings are consistent with other studies suggesting that leaders in healthcare organizations often choose a particular nursing care delivery system based on philosophy, available resources and contextual demands [47].

While the framework highlights a set of distinct factors that can shape different configurations of nursing care, our empirical analysis showed that staffing was particularly important in distinguishing unique clusters of nursing care organization models. Specifically, some organizational clusters had high intensity of nursing resources and/or a high proportion of nursing care hours provided by registered nurses, while others had low intensity of nursing resources and/or a low proportion of nursing care hours provided by registered nurses. One model was distinguished by a high proportion of nursing care hours provided by university-trained registered nurses. This may reflect the focus on staffing in many recent efforts to redesign nursing care. This finding was also consistent with many recent studies that have demonstrated the importance of staffing in designing nursing care delivery practices that guarantee best outcomes for patients.

In contrast, we found that inpatient medical nurses’ actual scopes of practice were quite constrained relative to their possible scopes. Furthermore, we found that units in each of the four models did not clearly differ on scope of practice for professional nurses. With regard to the work environment, the differences among the four models were stronger in capacity for innovation than in practice environment measures (based on a measure embodying magnet hospital characteristics). The innovative professional model had much higher capacity for innovation scores in comparison with the three other models (which were indistinguishable on this variable). The two professional models showed a relative superiority in terms of practice environment but had score profiles lower than those seen in magnet hospitals, as measured in earlier studies [29]. This suggests that while these various elements (scope of practice, capacity for innovation, practice environment) may all be important, managers do not always draw upon them when attempting to redesign nursing care delivery models. Another hypothesis is that changes in variables such as staffing can be more easily and more quickly implemented and then have a more visible impact in shaping the configurations of nursing care organization models than would be changes related to nurses’ actual scope of practice or work environment, whose impact may take longer to be visible. It can also be argued that staffing is a bedrock on which other practice developments must be built.

It is important to recognize that considerable variation was found across the units within the same model groups as well as across them. Although the units forming each model shared similar labels, this did not imply they were strictly identical. As shown in Table 6, we found within a same model units that presented the same features on most dimensions examined but differed on at least one dimension. These differences were not only due to the nature of the study, which reproduced variations inherent to real-world observations, but also reflected the dynamic features of organizational models, the numerous influences to which they are submitted and the varying degrees of success in various organizations’ efforts to redesign nursing care. Another hypothesis is that some units that feature the characteristics of a given model may be transitioning toward another model, due to changes affecting one or more key parameters of the conceptual framework.

This research remains only a first step towards a more complete taxonomy that will reflect the diversity of nursing care organization configurations and their complexity. Several limitations should be highlighted. First, these results relate primarily to medical units. Because the sample was selected so as to cover a wide range of contexts with regard to medical units, it is very likely that the taxonomy emerging from this analysis would capture the most prevalent models of organizing nursing care in the current context of medical units or other care sectors with similar features. For other sectors, although the proposed framework remains valid to examine models of nursing care, it may result in other combinations of the parameters considered. Similarly, because all the units selected were in Quebec, they may reflect the organization of the Quebec health care system and not capture specific features that may prevail in other jurisdictions. Second, the reality of nursing care organization models is immensely complex. To study them, we inevitably had to reduce the actual and diverse reality to a relatively small number of features, dimensions, and categories. Part of the reality may have been lost in this inevitable reduction process. Third, several of the measurement tools used in this study are based on nurses’ perceptions (scope of practice, work environment, capacity for innovation) and could be supplemented in future research with ethnographic techniques in order to deepen insight into those aspects of nursing care organization models. This means the taxonomy can and should be continually validated and refined, not only to take into account the distinct configurations driven by different contexts of care, but also to keep pace, within a given context, with rapid changes in nursing workforce, actual scope of practice and work environments.

## Competing interests

The authors declare they have no competing interests.

## Authors’ contributions

The study was conceived and designed by CAD and DD. All authors made a substantive contribution to all parts of the study: tool development, data collection and data analysis. CAD prepared the first draft of this manuscript. All authors contributed substantively to revising the manuscript. All authors read and approved the final manuscript.

## Pre-publication history

The pre-publication history for this paper can be accessed here:

http://www.biomedcentral.com/1472-6963/12/286/prepub
